# Model experiment research on HPTL anchoring technology for coal-rock composite roof in deep roadway

**DOI:** 10.1038/s41598-023-29232-5

**Published:** 2023-02-10

**Authors:** Zhengzheng Xie, Yongle Li, Nong Zhang, Zhe He, Chuang Cao, Wei Li

**Affiliations:** 1 State Key Laboratory of Coal Resources and Safe Mining, School of Mines, China University of Mining and Technology, Xuzhou, 221116 Jiangsu China; 2 School of Civil Engineering, Xuzhou University of Technology, Xuzhou, 221018 Jiangsu China; 3 Shandong Xinkuang Zhaoguan Energy Co., Ltd, Dezhou, 251113 Shandong China

**Keywords:** Engineering, Civil engineering

## Abstract

Since the western region of China, which is typical of extraordinary resource endowments, has gradually emerged as the major mining zone in China, the mining of thick coal seams and roadways with coal-rock composite roof have become more and more common in this region. However, it is extremely difficult to realize safe and effective maintenance and control of such roadways due to the differences in natural endowments of coal-rock masses. With the roadway with coal-rock composite roof of Hulusu Coal Mine in western China as the engineering background, experiment research on large-scale similarity model was conducted through comprehensive measures such as the pneumatic loading system, the surrounding rock stress monitoring system, the roadway deformation monitoring system, the bolt load monitoring system, and the displacement field monitoring system in this paper. According to the results of the experiment, the control effects of the three support systems on the roadway with coal-rock composite roof were significantly different. When the single support of short anchor bolts was applied, the comparatively low initial anchor-hold failed to constrain the initial micro deformation of the roof. Consequently, wide-range fractures of the roof were triggered at a loading pressure of 0.8 MPa. In the meanwhile, the deep surrounding rocks witnessed a downward inflection point in stress, accompanied by the possibility of the collapse of the thin-layer anchorage zone at any time. As for the support combining both short anchor bolts and long anchor cables, though a reinforced effect on the bolt anchorage zone could be achieved with the help of the cables, the active reinforcement capacity of the bolt was limited. The bolt anchorage zone was the first to be damaged at a loading pressure of 0.9 MPa, which would subsequently affect the effective bearing capacity of the deep surrounding rocks. In the application of the single support of high-strength long anchor bolts, the long bolts with high pre-tightening force were able to lock multiple groups of coal-rock strata to form a thick-layer anchorage bearing structure capable of withstanding a load as high as 1.0 MPa. The crash and collapse of the coal wall eventually caused the subsidence of the roof. Based on the intense dynamic load experiment and the feedbacks of engineering application outcomes in the field, it was concluded that the high-pretension thick-layer (HPTL) anchoring technology can effectively constrain the deformation of roadways with coal-rock composite roof with favorable application outcomes.

## Introduction

The coal mining activities in China are mainly concentrated in western provinces such as Shanxi, Shaanxi and Inner Mongolia, with most of the coal seams mined in these provinces having a thickness of 3.5 m^1,2^. Due to the limitations of mining height, the reservation of top coals is usually common during roadway excavation, and the rock strata above the top coal usually cover transition sections of mudstone or sandy mudstone that are lithologically poor and categorized as the composite roof^3^. Therefore, the roadways containing top coals are usually defined as the roadway with coal-rock composite roof. Subjected to the influences of coal-forming conditions, the coal usually consists of three types of structural planes^4–6^, namely the layering, the face cleat, and the butt cleat, all of which can reduce the mechanical properties of the coal. In the meanwhile, the fragmentation of the coal is more developed due to the influences of tectonic stress, which explains the differences in mechanical properties between the coal and the rock mass. According to a comprehensive analysis based on literature^7,8^, the roadway with coal-rock composite roof is typical of the following deformation characteristics: ① The coal seam differs significantly from the rock stratum in properties, accompanied by a higher possibility of the separation and collapse of the coal seam from the rock stratum after roadway excavation is started. ② Since cross cracks are widely distributed in the coal, the deformation of the surrounding rocks denotes the propagation of the cracks, which will easily lead to the embrittlement and fragmentation of the coal seam. ③ Since both the roof and the wall are coal, the crack propagation is transmissible and can thus cause the risk of wall fragmentation. To sum up, deep roadways with coal-rock composite roof are widely distributed in western China. Since it is difficult to achieve effective maintenance and control of the roof-wall linkage failure, it will be of great significance for China’s economic development in the future if such roadways are well under control.

Numerous scholars have conducted relevant research to address technological challenges concerning safe maintenance and control of roadways with coal-rock composite roof^9,10^. The exploration is performed firstly with interpretation of the failure mechanism of the roadway with coal-rock composite roof. The stress of roadway surrounding rocks changes from three ways to two ways or one way after excavation. Consequently, it is easy for shear stress to concentrate at the humeral angle of the roadway, leading to the propagation of the composite cracks in the top coal at higher odds. In addition to the formation of fragmentation in the roof as a result, even instability across a wide range might occur in the roof. As pointed out by some scholars^11^, for roadways with coal-rock composite roof, the shear slip failure takes place firstly in the coal wall under the action of the load, which will subsequently trigger the appearance of abscission layers and the falling-off failure in the roof.

The existing control methods for roadways with coal-rock composite roof mainly cover the following three technologies: ① Intensive support technology^12^. With this technology, it is viable to maintain the integrity of surrounding rocks by improving the initial strength of the support system, so as to avoid the occurrence of harmful deformation in surrounding rocks to the greatest extent. ② Truss-cable combined support technology^13,14^. The bolt support is only capable of providing radial force to the anchored rock mass, without the provision of tangential force. As a result, it is easy for the anchorage body in the roof to experience tangential displacement when the roadway roof happens to be the coal seam, making it difficult for the formation of a stable anchoring structure. On the other hand, the anchoring point in the truss cable is located in the deep rock mass at the humeral angle of the roadway, which has a larger action range and can effectively wipe out the abscission layer in the roof. Therefore, this technology is more effective in the control of the coal-rock composite roof. ③ Anchoring-grouting combined control technology^15,16^. Since the fragmentation failure is common in coal-rock composite roofs, their surrounding rocks are usually poor in anchorage. With this technology, however, grout can be injected into different types of cracks or weak planes of surrounding rocks to cement rock masses together. In this manner, the integrity of surrounding rocks can be enhanced, alongside the improvement of the mechanical parameters and deformation patterns of surrounding rocks. With full and comprehensive exploration, the above technologies focus on support strength, anchoring structures and surrounding rock modification, respectively, with favorable application outcomes in practice. However, for stoping roadways that only have a service life of 1–2 years, the truss-cable support technology and the anchoring-grouting combined control technology involve relatively high investment costs, leading to their less popularization in field application. Coupled with their technological processes that are comparatively complex, roadway excavation is conducted with low efficiency when such technologies are applied. In the meanwhile, the intensive support technology has failed to thoroughly eliminate the existing problems. Therefore, innovations are stilled called for in the maintenance and control technology for roadways with coal-rock composite roof.

According to certain geometric and physical relations, the model experiment had been accepted by many scholars to replace the field prototype. This method was not limited by external conditions, but also conducive to grasp the internal relationship behind the phenomenon. He et al.^17^ and Wang et al.^18^ made use of large-scale model experiments to propose a new mining method called automatically formed roadway (AFR) without advance tunnelling, and the bolt load in the model experiments could be used to feedback the roof stability state. With the roadway with coal-rock composite roof of Hulusu Coal Mine, Inner Mongolia, as the engineering background, a similarity model experiment was performed in this paper to compare the maintenance and control effects of different support systems on the roadway and explore the most efficient and optimal form of support technology for roadways with composite roof.

## Materials and methods

### Experimental materials

#### Similar simulated materials

The selection and performance test of similar materials play a key role in reflecting the evolution characteristics and deformation laws of the cracks in surrounding rocks of deep roadways with composite roofs^19,20^. In this model experiment, Haulage Roadway 21,205 of Hulusu Coal Mine was selected as the engineering background. The roadway adopted was a rectangular, with the section of a size of 5400 × 3200 mm and at an average burial depth of 620 m. As a roadway with coal-rock composite roof, Haulage Roadway 21,205 covers rock strata mainly composed of coal, argillaceous sandstone, siltstone, and fine sandstone.

Similar materials are generally made up of cementitious materials and aggregates. In terms of specific principles, the cementitious materials solidify and bond the aggregates through chemical reactions, during which the properties of cementitious materials determine the plasticity and brittleness characteristics of similar materials directly^21^. High strength can be achieved after the setting and solidification of cement-type cementitious materials. However, apparent brittleness characteristics exist, alongside comparatively low strength, after the setting and solidification of gypsum-type cementitious materials. Therefore, similar materials made up of cement materials and gypsum materials in a certain proportion are typical of specific strength and brittleness, making them proper for simulating most coal rock masses in coal measure strata. To sum up, the material mixed with cement and gypsum was selected as the cementitious material and quartz sand as the aggregate in this model experiment.

Because the size of the roadway section must match reasonably with the overall size of the model and the size of the experimental system in the experimental model, the geometric similarity ratio was determined as $C_{l} = L_{p} {/}L_{m} = 20:1$. In this way, the deformation characteristics of the surrounding rock mass of the roadway can be characterized accurately. Since the average density of the rock mass in the field was tested as 2.5 g/cm^3^, the average density of the simulated rock mass was set as 1.65 g/cm^3^, the density similarity ratio as $C_{\rho } = \rho_{p} /\rho_{m} = 50:33$, and the stress similarity constant as $C_{\sigma } = C_{l} \cdot C_{\rho } = 1000:33$, respectively. As a result, the similarity constants of tensile strength $\sigma_{t}$, compressive strength $\sigma_{c}$, flexure strength $\sigma_{f}$, shear strength $\sigma_{s}$ and elasticity modulus $E$ of the rock mass all stood at 1000:33. As revealed by the geological occurrence of Hulusu Mine, the compressive strength of coal, siltstone, sandy mudstone and fine sandstone was tested as 9–15 MPa, 45–48 MPa, 30–33 MPa and 38–44 MPa, respectively, so the simulated strength of coal, siltstone, sandy mudstone and fine sandstone was calculated as 0.3–0.6 MPa, 1.5–1.6 MPa, 1.0–1.3 MPa and 1.2–1.4 MPa, respectively, based on the similarity ratio.

**Figure 1:**
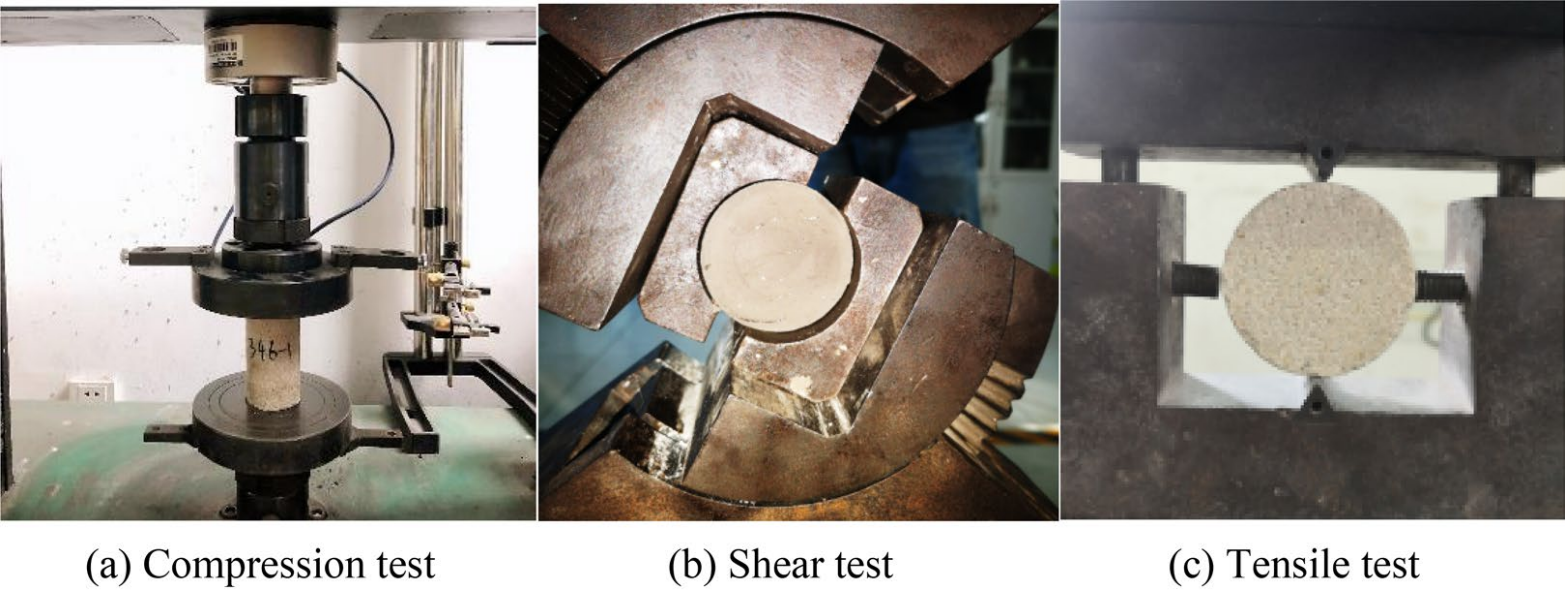
Experiment on mechanical properties of similar materials.

To prepare samples with different strength, four sand-to-binder ratios (3:1, 4:1, 5:1, 6:1 and 7:1) and three cement-to-gypsum ratios (3:7, 4:6 and 5:5) were applied pursuant to the scope of simulated strength of different rock strata. Specifically, 15 groups of standard cylindrical samples of Ф50 × 100 mm, 15 groups of standard cylindrical samples of Ф50 × 50 mm, and 15 groups of standard cylindrical samples of Ф50 × 25 mm in size were prepared for independent compression test, shear test and tensile test, respectively, with each group containing three samples for the test (Fig. 1). According to comparisons between different groups in the experiment, the proportioning ratio of similar materials among coal, siltstone, sandy mudstone, and fine sandstone were eventually decided as 737, 646, 637 and 755 (for instance, the proportioning ratio of 737 represents a sand-to-binder ratio of 7:1 and a cement-to-gypsum ratio of 3:7). Table 1 presents the mechanical parameters of the similar materials selected in the experiment.

**Table 1:** Proportioning number and mechanical parameters of similar rock strata.

Rock stratum lithology	Proportioning ratio	Uniaxial compressive strength /MPa	Elastic modulus /GPa	Tensile strength /MPa	Cohesion /MPa	Internal friction angle/°
Coal	737	0.58	0.32	0.10	0.17	27.5
Sandy mudstone	637	1.18	0.54	0.22	0.21	29.3
siltstone	646	1.57	0.83	0.30	0.29	29.8
Fine sandstone	755	1.28	0.68	0.18	0.24	32.4

#### Experimental anchor bolts

Three identical roadways were excavated in the model experiment to compare and verify the maintenance and control effects of different support systems for the roadways with the composite roof. Furthermore, the three roadways were subjected to three different support patterns, *i.e.*, the support system combining both anchor bolts and cables, the single support system with short anchor bolts, and the single support system with long anchor bolts. The support items adopted in the experiment comprised thread steel anchor bolts, high-strength long anchor bolts, and anchor cables. Nevertheless, very few bolts, cables, and other similar materials fully met the similarity relations required, and the similar bolts that could exert a comparatively large pre-tightening force were less. Using metal screws to simulate bolts and cables, some scholars believe that it would be easier for stainless steel screws to exert pre-tightening forces with different amplitude.

**Figure 2:**
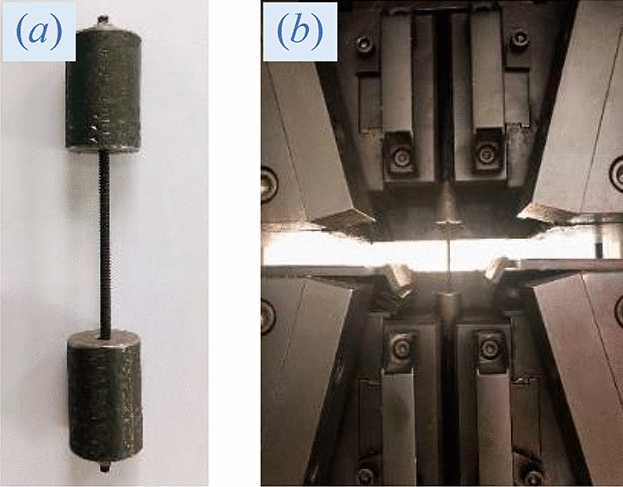
Mechanical property test of experimental bolt: (**a**) experimental bolt and pattern die, (**b**) detail of bolt drawing test.

Therefore, stainless steel screws and carbon steel screws were adopted for the test, with the test photos shown in Fig. 2 and the specific properties and specifications in Table 2. It was concluded that the Ф3-mm stainless steel screw had an average peak load of 3839 N and an elongation of 19.7%, which was consistent with the characteristics of the bolt; and the Ф3-mm carbon steel screw possessed an average peak load of 4727 N and an elongation of 2.8%, fitting the characteristics of the high-strength long bolt and the cable. As a result, the Ф3-mm stainless steel screw was used as the bolt, with the Ф3-mm carbon steel screw as the high-strength long bolt and the cable.

**Table 2:** Performance parameters of experimental bolts.

Type	Specifications	Peak force/N	Average peak force/N	Average elongation/%
Stainless steel screw	Ф3 × 250 mm	3995/3667/3855	3839	19.7
Carbon steel screw	Ф3 × 250 mm	5019/4601/4562	4727	2.8

**Figure 3:**
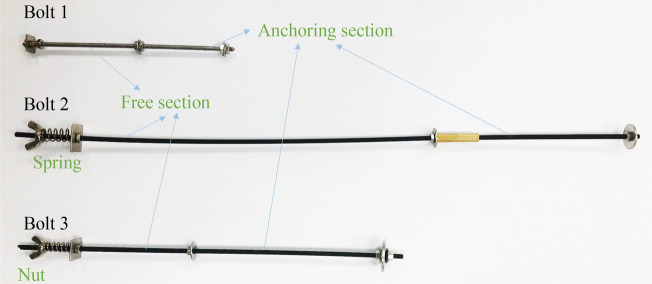
Experimental bolt.

A similar bolt comprised a screw, a gasket, a compression spring, and a bolt nut. As shown in Fig. 3, it consisted of a bolt, a cable, and a high-strength long bolt, all of which were measured as 125 mm, 325 mm and 225 mm, respectively, in length from top to bottom. Each bolt extended 25 mm externally for easier exertion of the pre-tightening force by the bolt nut. The length of the anchoring section of the experimental bolt was determined by the gasket, and the gap between the gaskets was anchored by cement mortar.

### Experimental system

#### Loading system of the model

The large-scale similarity model loading system of the State Key Laboratory of Coal Resources and Safe Mining, China University of Mining and Technology was adopted in the similarity model experiment, with the model of 2.5 m (width) × 1.2 m (height) × 0.2 m (thickness) in size. A dimension of 50 (width) × 24 (height) × 4 (thickness) m can be simulated for the strata when calculated at a geometric similarity ratio of 1:20. The left, right and lower boundaries of the experimental model were all set as the horizontal displacement constraint while the upper boundary of the model was used as the in-situ stress field through air pump simulation, so as to provide a maximum loading pressure of 1.2 MPa for the model.

#### Surrounding rock stress monitoring system

**Figure 4:**
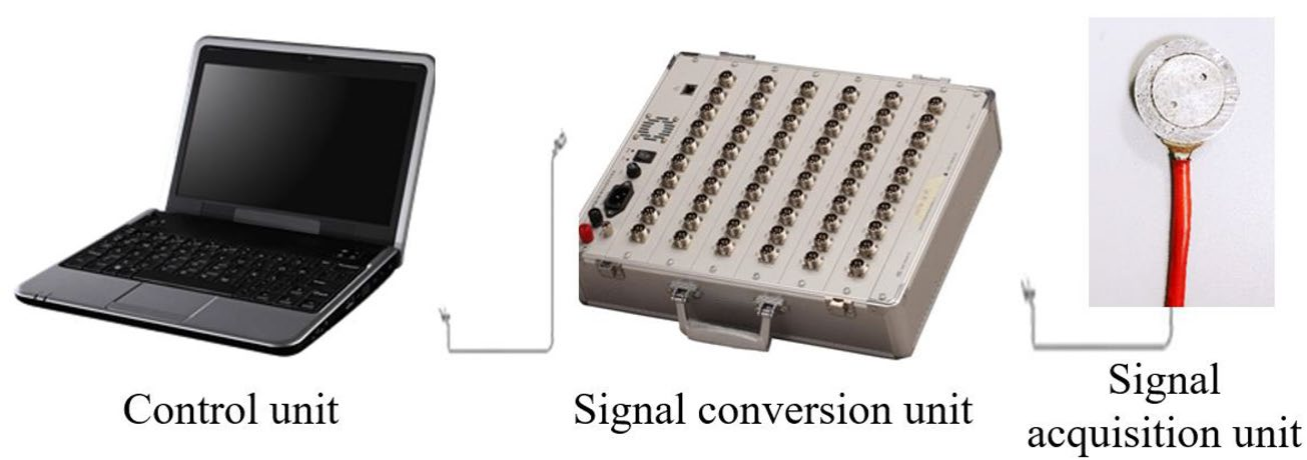
Static strain test system.

The evolution characteristics of the internal pressure of the model was monitored using the DH3816N static strain test system produced by Donghua Test, with the experimental system presented in Fig. 4. The static strain test system was composed of the control unit, the signal conversion unit, and the signal acquisition unit, serving as the software system, the static resistance strain indicator, and the pressure sensor, respectively. In addition, the static strain test system covered a total of 60 channels that could conduct real-time monitoring of 60 measuring points at the same time.

#### Roadway surface deformation monitoring system

**Figure 5:**
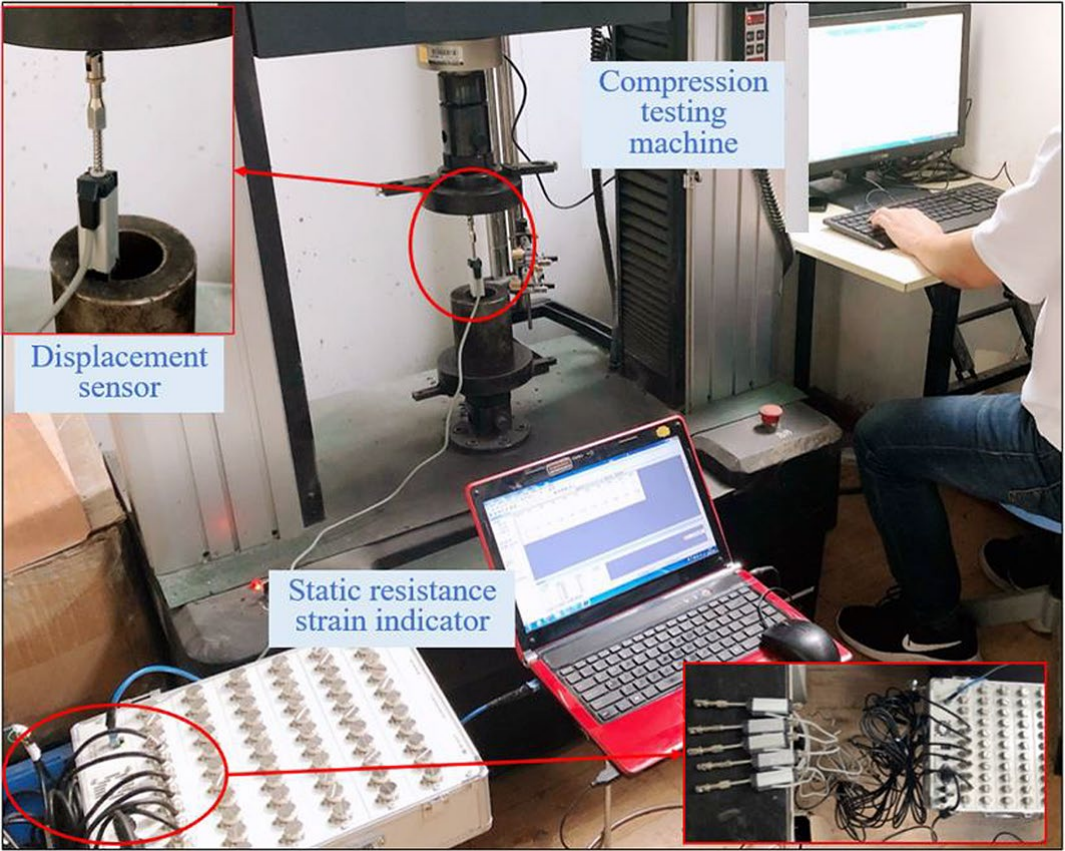
Displacement sensor and its calibration experiment.

**Figure 6:**
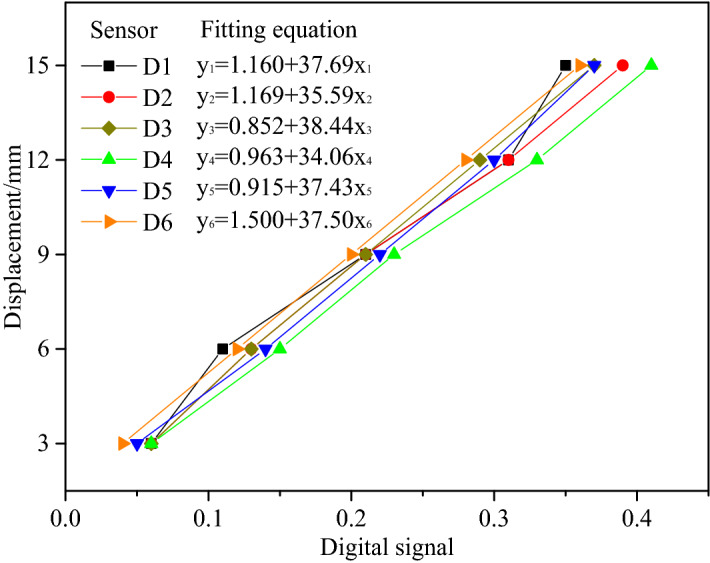
Data linear fitting and regression equation.

Real-time monitoring of roof subsidence of the roadway and convergence of the coal wall was conducted using a KTR2 displacement sensor, which had the maximum traveling distance of 15 mm and a measurement accuracy of ± 0.1% (Fig. 5). The displacement sensor and the static resistance strain indicator functioned together as the roadway surface deformation monitoring system, which was capable of converting displacement signals into digital signals. As presented in Fig. 6, roadway surface deformation was measured in an accurate manner by calibrating the quantitative relationship of the displacement sensor in the laboratory.

#### Bolt load monitoring system

**Figure 7:**
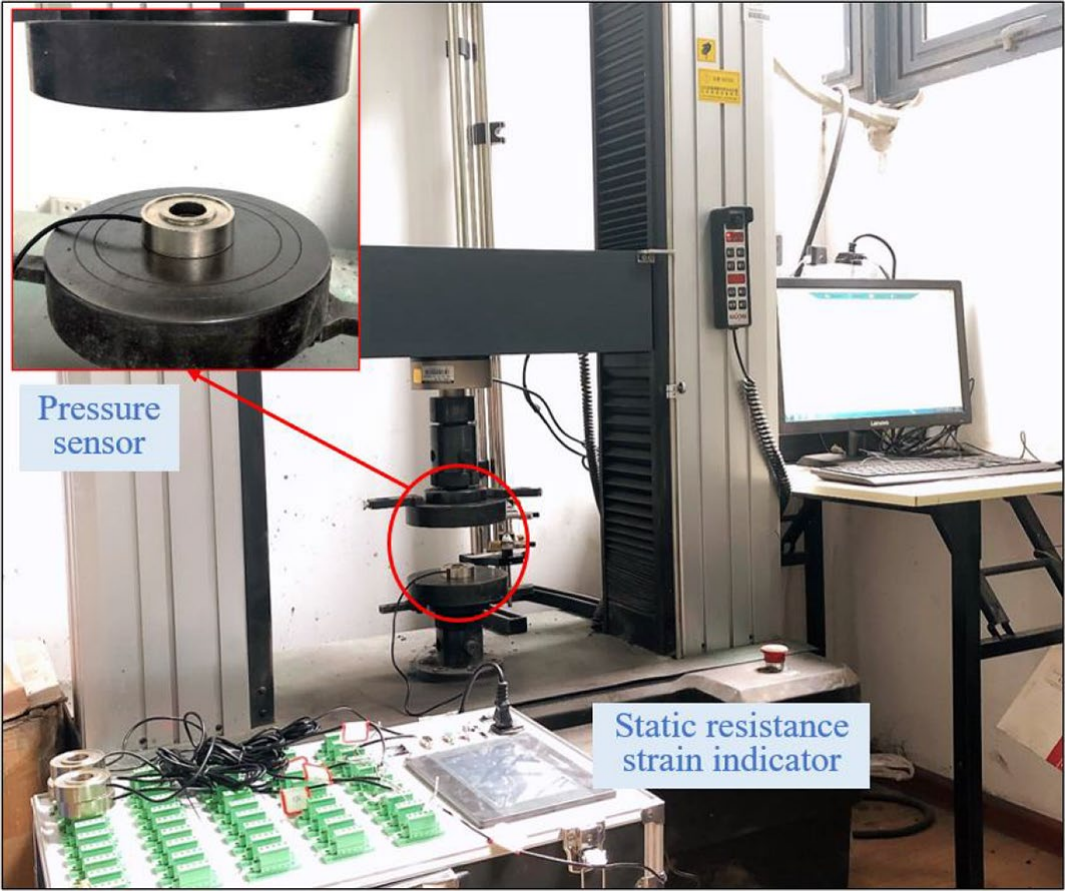
Force sensor and its calibration experiment.

**Figure 8:**
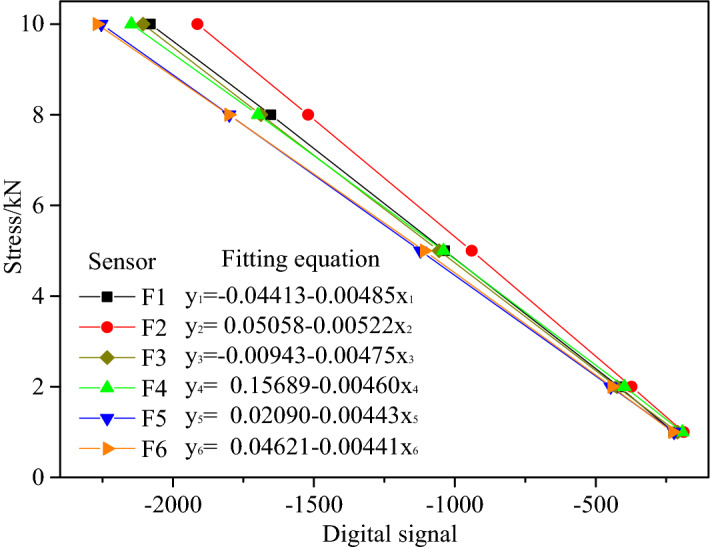
Data linear fitting and regression equation.

Real-time monitoring of the axial load of the similar bolt was performed using a force sensor (Fig. 7). The pressure sensor had a measuring range of 0–1 T with a measuring accuracy of ± 0.2%. The force sensor was calibrated using a pressure tester to acquire the corresponding relationship between the output digital signal and the load. Figure 8 presents the calibration experiment.

#### Monitoring system for surface displacement fields of surrounding rocks

The whole field displacement of the surrounding rock of the roadway was monitored using the digital speckle correlation measurement system, which was composed of the software unit and the camera unit. The speckle correlation measurement system (MatchID-2D) mainly works based on the following principle^22^: The images before and after the deformation of the object are acquired through the camera; then the software system is utilized to acquire the speckle gray characteristics on the images before and after the deformation; corresponding relations between different images are subsequently established, with corresponding points on the images before and after the deformation identified; and the displacement of each point in the object is determined in the end.

### Experimental model and program

#### Experimental model

**Figure 9:**
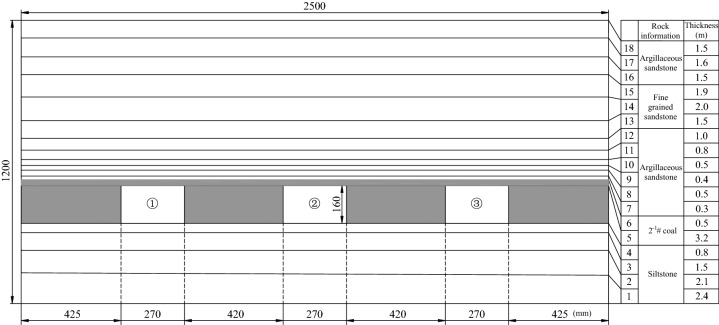
Model map of strata distribution.

Figure 9 presents the relations between the size of the experimental model, the distribution of the coal measure stratum and the location of the roadway. Three identical roadways, 270 (width) × 160 (height) mm in size, were arranged in the 2–1 coal seam, with a spacing of 420 mm between adjacent roadways. A 425-mm large coal pillar was reserved for the roadway on each side to mainly prevent stress interference and guarantee similar boundaries. Weak planes with cleavages and cracks distribute widely in the coal measure stratum. To well reflect the actual situation, the coal measure stratum was divided into multiple layers for laying of the model.

The stress environment of the model was designed in line with the occurrence conditions of Haulage Roadway 21,205. Since the mining coal seam is typical of 2–1 coal and buried at a depth of 620 m, the in-situ rock stress of this layer stood at 15.5 MPa, and the actual in-situ rock stress of the boundary above the model was tested as 15.2 MPa. Therefore, the in-situ stress field above the experimental model was calculated as 0.5 MPa based on the stress similarity ratio of 1000:33.

#### Roadway support schemes

**Figure 10:**
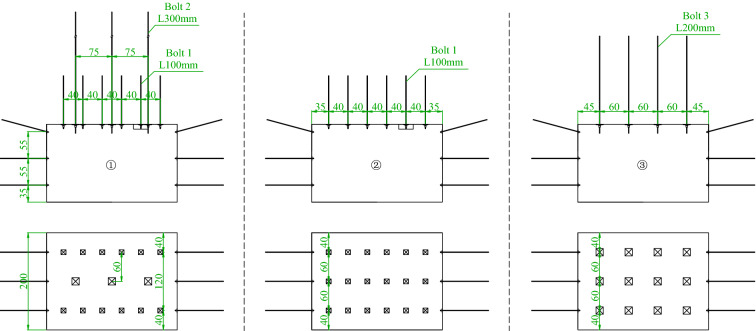
Design diagram of test roadway support.

The support combining short bolts and long cable, the single support with short bolts and the single support with high-strength long bolts were adopted for the roofs of Roadways ①–③, respectively, with the walls of the three roadways all supported by short bolts. Figure 10 presents the specific support parameters. Calculated based on the geometric similarity ratio, the cables and the bolts of Roadway ① were 300 mm long at a row spacing of 75 × 120 mm and 100 mm long at a row spacing of 40 × 120 mm, respectively; the bolts of Roadway ② were 100 mm long at a row spacing of 40 × 60 mm; and the high-strength long bolts of Roadway ③ were 200 mm long at a row spacing of 60 × 60 mm.

#### Layout of measuring points

**Figure 11:**
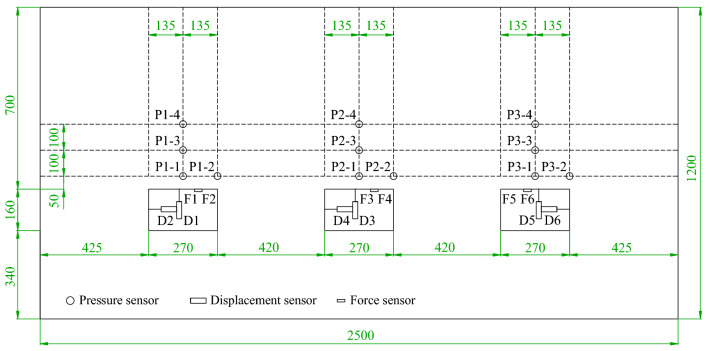
Sensor layout plan.

Surrounding rock pressure, bolt stress, roadway surface deformation, and surrounding rock surface displacement field of the model were monitored via the information acquisition system in a comprehensive manner to analyze the bearing mechanisms of coal-rock composite roofs supported by different anchorage systems from multiple perspectives. Pressure sensors were installed at the same positions on the roofs of the three roadways, with the layout of specific monitoring points shown in Fig. 11. Four pressure sensors were arranged above the roof of each roadway, with the pressure sensors P1 and P2 located in the layer 50 mm above the roof, the pressure sensor P3 in the layer 150 mm above the roof, and the pressure sensor P4 in the layer 250 mm above the roof. Two displacement sensors were equipped within each roadway to monitor the deformation in the middle of the roof and in the middle of the wall, respectively, with the displacement sensors coded as D1, D2… D6. Two force sensors were arranged on the roof of each roadway, with a sensor installed on both the bolt and the cable of Roadway ① (namely the force sensors F1 and F2), two sensors on the high-strength long bolt of Roadway ② (namely the force sensors F3 and F4), and two sensors on the short bolt of Roadway ③ (namely the force sensors F5 and F6).

#### Process of model making

**Figure 12:**
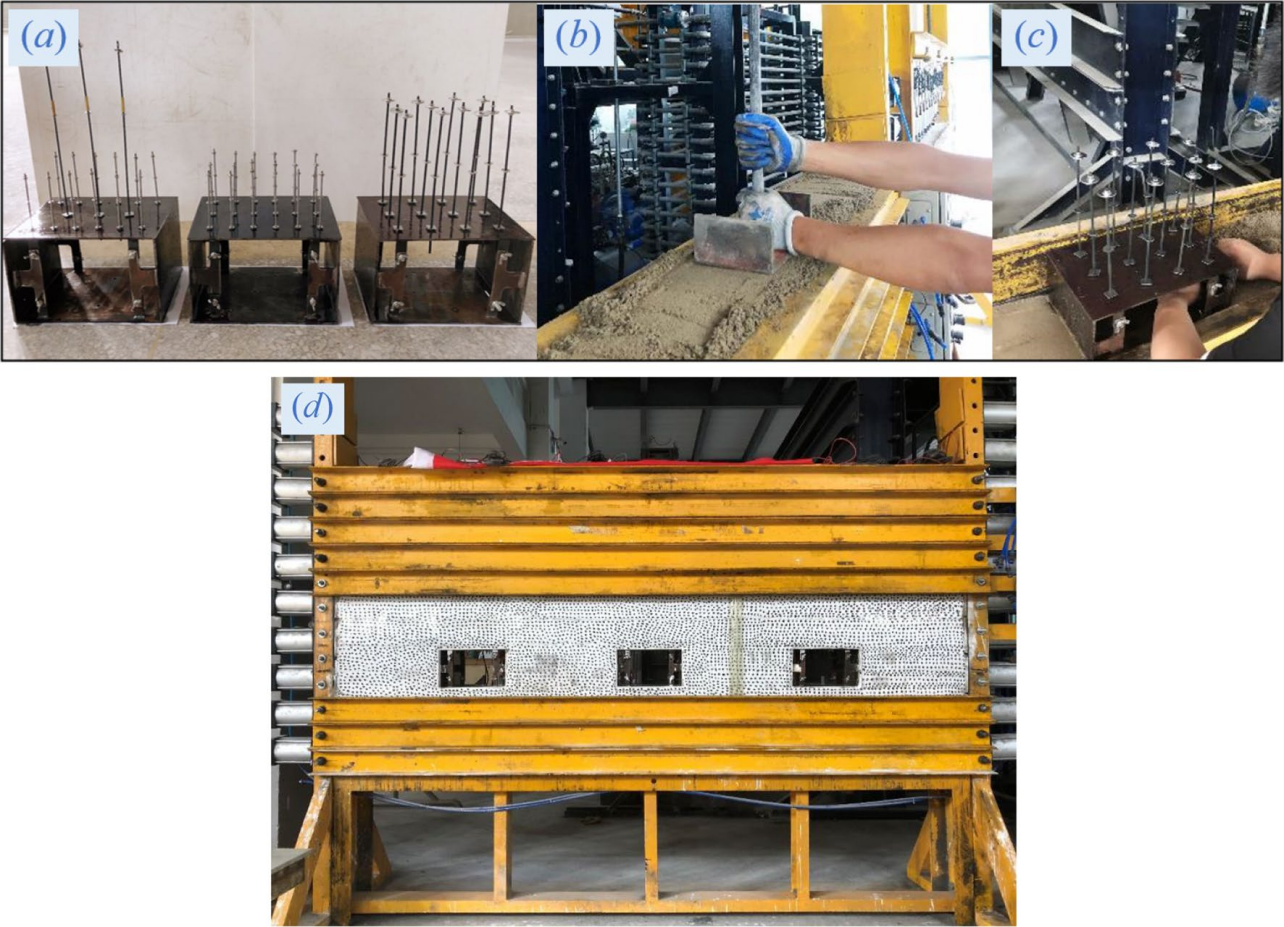
Main steps of model making.

The making of the experimental model mainly covered the following 6 steps: (a) The experimental bolts were assembled and installed into the model of the roadway in advance after the assembly. (b) Similar materials for various rock strata were prepared pursuant to the proportioning ratio listed in Table 1, and were poured into the model frame for tamping in line with the distribution of rock strata after they were prepared. (c) Roadway models and pressure sensors were embedded into the experimental model in accordance with their locations planned. (d) The baffle boards at the front and rear of the model were removed 7 days after the laying of the model; then the model was subjected to natural drying for another 20 days, followed by the treatment of whitewashing and painting with black spots on the model surface. (e) Baffle boards were installed at the front and rear of the model, and the observation areas around the roadways were extruded with transparent acrylic plates to apply stress constraints on the observation areas. Figure 12 presents the main steps of model making.

#### Debugging and testing of the model

**Figure 13:**
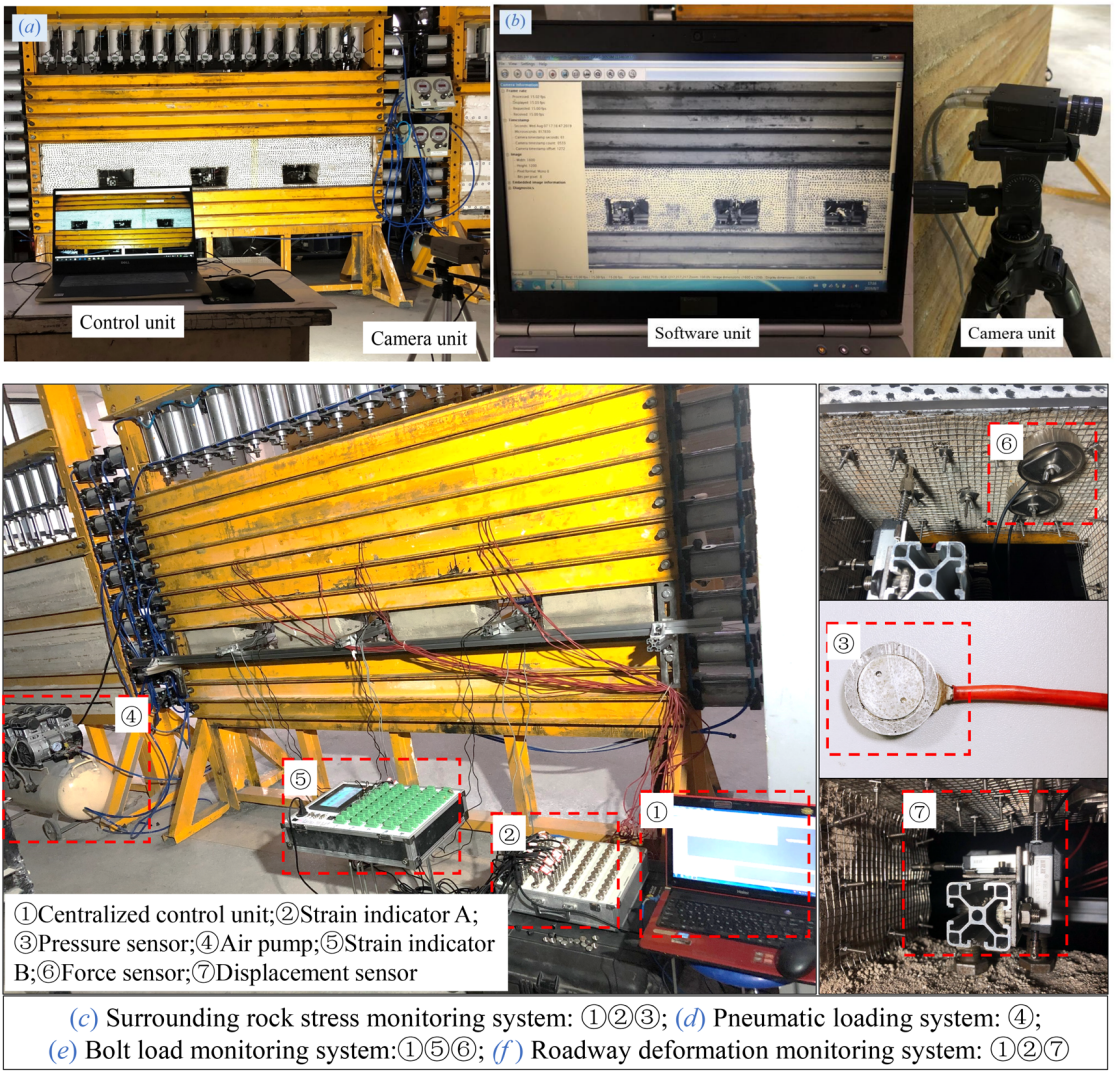
Each monitoring system of the experimental model.

The preparation, application of in-situ stress (Stage I), excavation and unloading of the roadway (Stage II), and progressive loading of the pressure (Stage III) constituted the debugging and testing process of the model, with the specific steps listed as follows:

1. Preparation: The most appropriate positions for image recording by the video monitoring system (Fig. 13a) and the whole field displacement monitoring system (Fig. 13b) were spotted, followed by the initiation of the automatic recording service. Next, the static resistance strain indicator was connected to the interfaces of the pressure sensors, the values of the pressure sensors were calibrated, and the surrounding rock pressure monitoring system (Fig. 13c) was initiated.
2. Application of in-situ stress: The pneumatic loading system was initiated (Fig. 13d) to apply the in-situ stress filed to the model in a gradual process. The pressure was loaded at 0.1 MPa, 0.2 MPa, 0.3 MPa, 0.4 MPa and 0.5 MPa, respectively, at five stages in a progressive manner, with a time interval of 20 min between each loading.
3. Excavation and unloading of the roadway: The support plates of each roadway were removed to complete the excavation of the roadway, followed by the installation of the metal mesh and the nuts of the experimental bolts in the roadway. Then the pre-tightening force was applied to the bolts. Two force sensors were installed on the roof of each roadway to calibrate the axial load of different bolts, with the initial pre-tightening force of the short bolt, the cable, and the high-strength long bolt set as 17.4 N, 37.2 N, and 59.4 N, respectively (Fig. 13e). The bearing brackets of the displacement sensors were subsequently installed before the displacement sensors were arranged pursuant to the design requirements (Fig. 13f), after which the calibration was performed.
4. Progressive loading of the pressure: The pressure above the model was increased in a gradual manner until the model was crushed, with the deformation and failure characteristics of each roadway observed and recorded. An amount of 0.1 MPa was added at each loading, with a time interval of 40 min between each loading.

## Experimental results and analysis

### Evolution of the stress of roadway surrounding rocks

**Figure 14:**
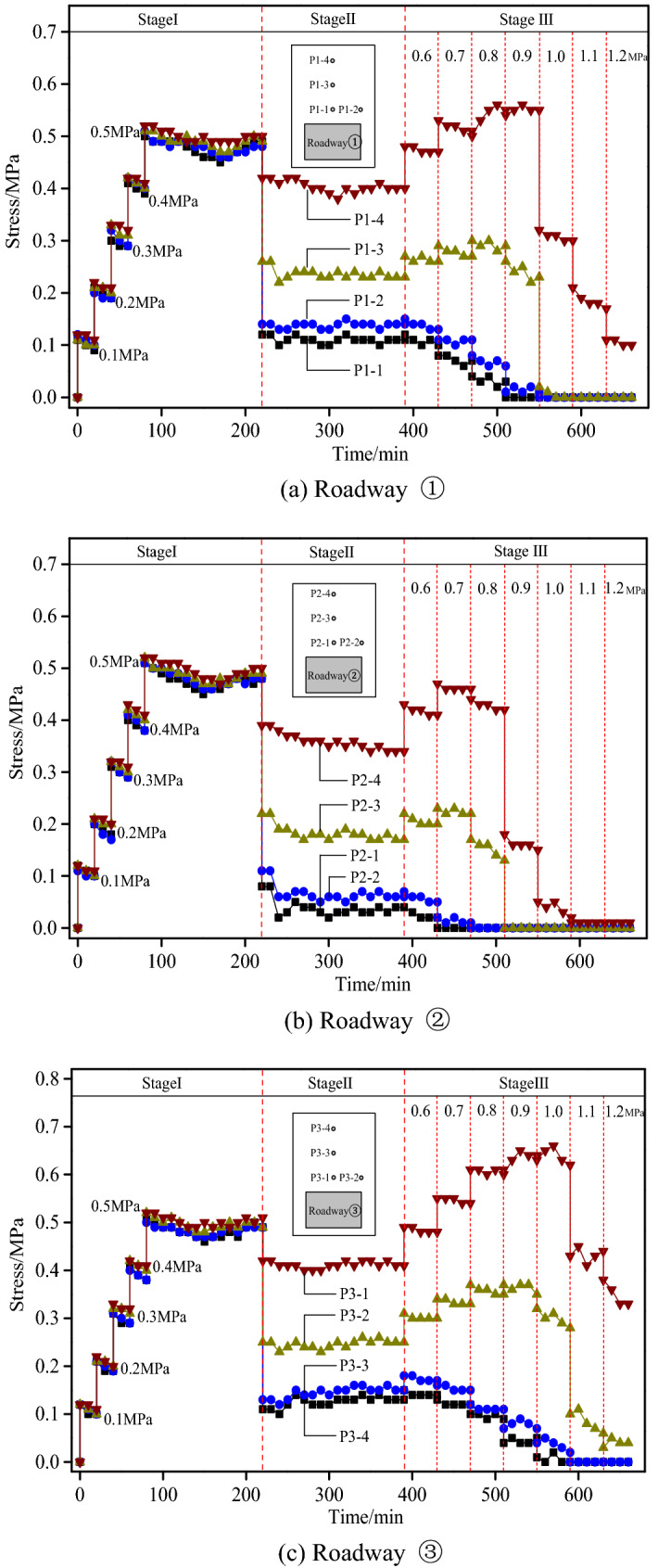
Stress evolution curves of roadway surrounding rocks.

Figure 14 presents the stress evolution curves of roadway surrounding rocks, revealing that as roadway excavation and model loading carried on, the evolution laws of the stress of surrounding rocks in each roadway exhibited both similarities and differences. The first stage witnessed similar stress evolution curves of the three roadways, all of which indicated a trend of gradient increase. the surrounding rock stress of each roadway dropped slightly with a tendency of remaining stable when the surrounding rock stress reached 0.5 MPa. In the second stage, the surrounding rock stress decreased sharply when the roadway was initially excavated, with the pressure sensors closer to the roof witnessing a larger drop while those further from the roof experiencing a smaller drop in stress value. Furthermore, the surrounding rock stress rose slightly and was subjected to dynamic adjustments after the bolt support was applied. In the third stage, significant differences occurred in the evolution of the stress of different layers as the loading pressure increased gradually. The evolution of the stress on the roofs of the three roadways will be discussed in independent sections.

The roof of Roadway ① was co-supported by short bolts and long cables. The sensors P1-1 and P1-2 were located within the bolt anchorage zone, while the sensors P1-3 and P1-4 were installed outside the bolt anchorage zone but still within the cable anchorage zone. According to Fig. 14a, the stress of P1-1 and P1-2 decreased gradually when the loading pressure rose slowly to 0.8 MPa, revealing the appearance of crack abscission layers firstly in the top coal during this process; but the sensors P1-3 and P1-4 witnessed the rise of the stress, indicating that no cracks developed outside the bolt anchorage zone under this pressure and only elastic–plastic deformation occurred in the deep surrounding rocks. The stress in the bolt anchorage zone almost dropped to 0 when the pressure rose to 0.9 MPa, implying the generation of comparatively large abscission layers in the bolt anchorage zone and the separation of the top coal from the deep rock strata; at this moment, the stress of the sensor P1-3 had begun to drop with a descending trend, indicating the appearance of abscission layer damages in the tail area of the bolt, as well as the occurrence of crack propagation outside the bolt anchorage zone; moreover, the stress of the sensor P1-4 started to fluctuate. The entire bolt anchorage zone collapsed when the loading pressure reached 1.0 MPa, resulting in a sudden drop of the stress of roof surrounding rocks; at this moment, the stress of the sensor P1-3 dropped to 0 while that of the sensor P1-4 still remained above 0, indicating that the deep rock mass still retained its bearing capacity to some degree.

In Roadway ②, the roof was under single support of short bolts. The sensors P2-1 and P2-2 were located within the bolt anchorage zone, while the sensors P2-3 and P2-4 were installed outside the bolt anchorage zone. According to Fig. 14b, the stress of both sensors P2-3 and P2-4 rose when the loading pressure reached 0.6 MPa and 0.7 MPa, respectively, with the sensor P2-4 exhibiting a larger amplitude of increase. The stress of the sensors P2-1 and P2-2 dropped, however. The stress of the sensor P2-1 decreased to 0 directly at a pressure of 0.7 MPa, denoting that the cracks between the top coal and the upper rock stratum had run through to form abscission layers. Given a pressure of 0.8 MPa, the surrounding rock stress of the sensors P2-3 and P2-4 both dropped slightly, indicating the propagation of the cracks to deeper strata at this moment. The bolt anchorage zone instantly collapsed when the loading pressure reached 0.9 MPa, due to which the stress in the deep stratum was suddenly released. Consequently, the stress of the sensors P2-3 and P2-4 dropped in a gradient manner.

The roof of Roadway ③ was under single support of high-strength long bolts, for which the sensors P3-1, P3-2 and P3-3 were installed within the bolt anchorage zone while the sensor P3-4 outside the bolt anchorage zone. As shown in Fig. 14c, when the loading pressure rose to 0.9 MPa in a gradual process, the sensors P3-1 and P3-2 witnessed a slight decrease while the sensors P3-3 and P3-4 a gradual increase in stress. This indicated that though cracks had started to develop in the top coal area of the bolt anchorage zone as the pressure rose, the bolt anchorage zone still functioned with effective bearing capacity at this moment. This further proved that thick anchoring layers of the roof were able to resist a large amount of load. The development of surrounding rock cracks became active when the loading pressure reached 1.0 MPa, with the sensor P3-3 witnessing a drop while the sensor P3-4 not yet a decline in stress. This indicated that the cracks had not yet developed to this location. The walls were crushed at a loading pressure larger than 1.0 MPa, resulting in the overall subsidence of the roof and the generation of abscission layers between rock strata. Consequently, the stress of surrounding rocks dropped in a gradient manner. At this moment, however, the roof still remained comparatively intact, with the existence of safety spaces in the roadway to some degree.

### Evolution of bolt axial load

**Figure 15:**
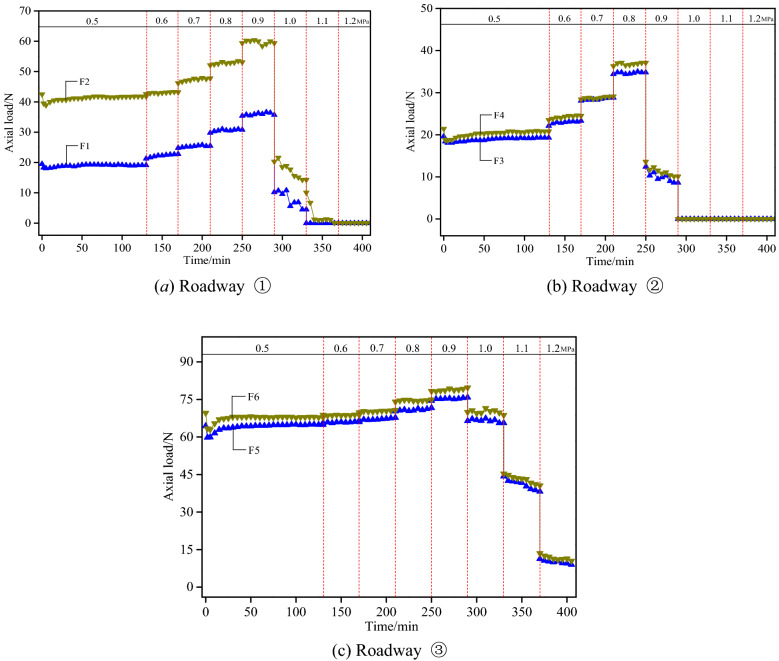
Variation curves of axial load of experimental bolt.

The evolution curves of the axial load of the experimental bolts are presented in Fig. 15, which reveals the existence of significant differences in the evolution of the axial load of the bolts in each roadway.

The force sensors F1 and F2 were installed on the bolt and the cable, respectively, as shown in Fig. 15a. The axial load of the bolt and the cable were 19.5 N and 42.5 N, respectively, shortly after the pre-tightening force was applied; and the load of the bolt/cable dropped slightly after the bolt nut was loosened, with a reduction rate of 7.2% and 8.5%, respectively. This denoted the existence of load rebound in the experimental bolt after the pre-tightening force was applied. Then slight deformation occurred in the roof, during which the axial load of the bolt and the cable slightly increased in fluctuation. At a load of 0.6 MPa, deformation took place firstly in the top coal, for which the bolt performed to exert a timely restraining effect. At this moment, the load of the force sensor F1 rose sharply to reach 11.5%; accordingly, the corresponding deep surrounding rocks experienced deformation that was relatively small, and the load of the force sensor F2 increased only by 1.9%. When the load rose from 0.7 MPa to 0.9 MPa, the deformation of surrounding rocks extended by degrees as the loading pressure gradually increased. Since the bolt and the cable played a key role in the provision of active bearing support, the force sensors F1 and F2 both witnessed a sharp rise of axial load. Cracks occurred in the bolt anchorage zone when the load exceeded 0.9 MPa, causing a sudden drop of the axial load of both the bolt and the cable. The axial load of the bolt/cable gradually dropped to 0 as the loading pressure further increased.

The force sensors F3 and F4 were both installed on the short bolt, as revealed in Fig. 15b. The initial load of the bolt measured by the two force sensors was 19.6 N and 21.4 N, respectively. The axial load of the bolt exhibited a trend of progressive increase when the load rose from 0.5 MPa to 0.8 MPa by degrees. As the deformation of surrounding rocks aggravated, the rising amplitude of the load gradually enlarged. At a load of 0.8 MPa, the maximum axial load of the bolt reached 35.0 N and 37.1 N with an increase by 78.6% and 73.4%, respectively, according to the two sensors. This revealed the active role of the short bolt in controlling the deformation of surrounding rocks. The comparatively short length of the bolt, however, resulted in the smaller thickness of the anchoring layer. Consequently, it would be easy for the cracks to propagate beyond the anchorage zone. When the load was larger than 0.8 MPa, the cracks outside the anchorage zone quickly ran through to trigger collapse of the roof, accompanied by the drop of the bolt load to 0.

According to Fig. 15c, the force sensors F5 and F6 were both installed on the high-strength long bolt, with the initial load of the bolt measured by the two force sensors as 64.4 N and 69.6 N, respectively. After the release of the wrench, the bolt load dropped slightly, increased rapidly, and finally became stable. This process of evolution denoted that in the event of minor deformation in the top coal, the bolt with high pre-stressing force could function immediately to control the deformation of surrounding rocks. Progressive increase occurred by degrees in bolt load when the load gradually rose to 0.9 MPa. Compared with Roadways ① and ②, however, the axial load of the medium and long bolts in Roadway ③ increased at a significantly reduced amplitude. This characteristic also revealed the effective maintenance of the integrity of the roof and the result that the cracks did not develop widely under the action of the high-strength long bolt. The maximum load was tested as 75.8 N and 79.7 N, respectively, at this moment. The top coal was slightly damaged when the loading pressure reached 1.0 MPa, leading to a slight drop of the axial load of the bolt. The load was measured as 66.4 N and 69.9 N with a reduction rate of 12.4% and 12.3%, respectively. At a loading pressure of 1.1 MPa, the cracks propagated further upward to generate lateral abscission layers. On the other hand, because of the comparatively low strength of the coal, the walls collapsed under the action of high stress to trigger overall subsidence of the roof. As a result, the axial load of the bolt dropped significantly with a reduction rate of 42.5% and 41.9%, respectively.

To sum up, the evolution of the axial load of the bolt and the cable reflected the maintenance status of the roof surrounding rocks that are indirectly anchored by different support systems. Minor deformation first occurred in the top coal at the initial stage after the bolt support was applied, during which the bolt and the cable functioned immediately to increase the axial load. During this process, a greater pre-tightening force contributed to bolt responses that were more sensitive and acted timelier. As the loading pressure increased progressively, significant differences occurred in the variation laws of the axial load of different bolts and cables. From the perspective of variation amplitude of axial load, Roadway ② > Roadway ① > Roadway ③. In terms of resistance ability of surrounding rocks against deformation, however, Roadway ③ > Roadway ① > Roadway ②.

### Evolution of roadway surface displacement

**Figure 16:**
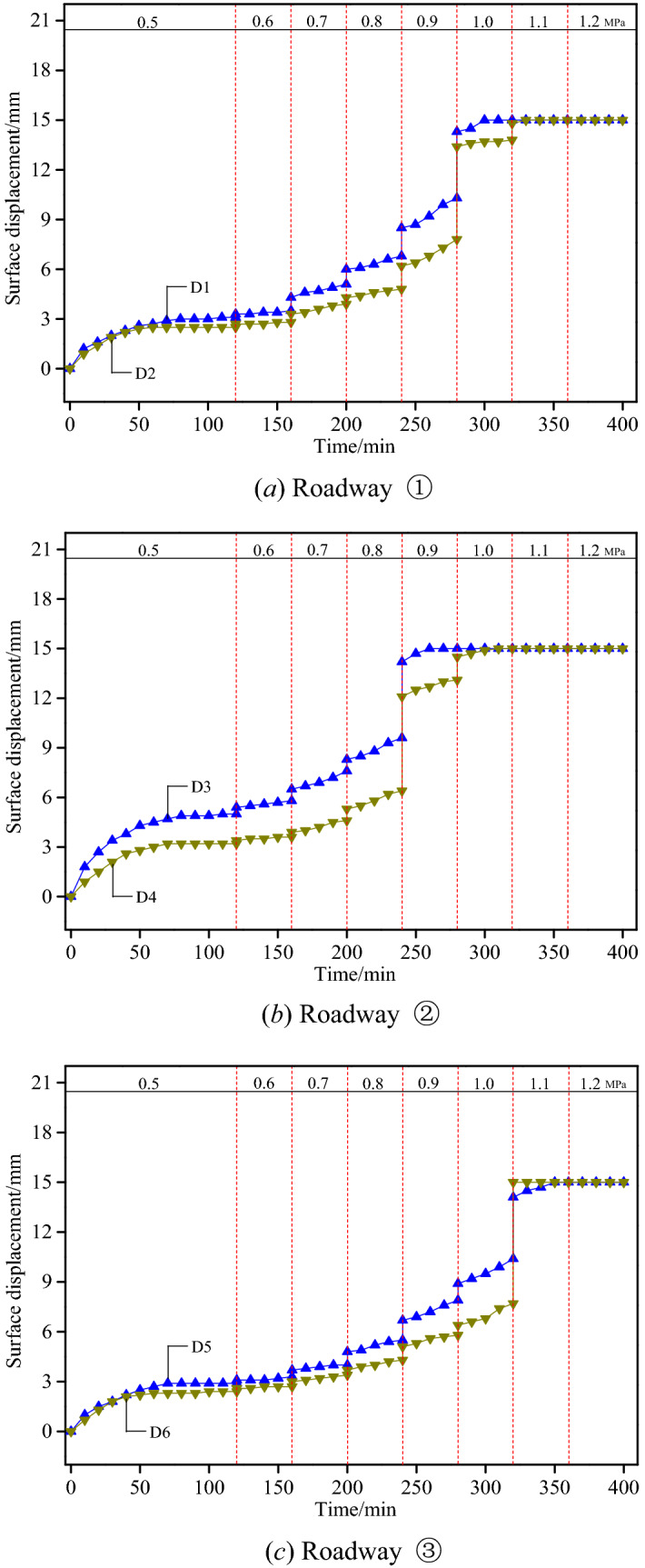
Surface displacement variation curves of roofs and walls of various roadways.

Figure 16 presents the surface displacement evolution curves of the roofs and walls of various roadways, based on which different evolution characteristics of each roadway are revealed.

According to Fig. 16a, the displacement of the roof and the coal wall of Roadway ① was measured by the displacement sensors D1 and D2, respectively. At a loading pressure of 0.5 MPa, the roadway surface displacement increased rapidly and then slowly and finally became stable. The subsidence of the roof and the convergence of the coal wall amounted to 3.1 mm and 2.5 mm, respectively. Little deformation occurred in surrounding rocks when the loading pressure reached 0.6 MPa, with an increase of roof subsidence and coal wall convergence only by 0.4 mm and 0.3 mm, respectively; at a loading pressure of 0.7 MPa, 0.8 MPa and 0.9 MPa respectively, the deformation of surrounding rocks was featured with a rapid increase by leaps, with the roof subsidence amounted to 5.1 mm, 6.8 mm and 10.3 mm and the relative displacement to 1.6 mm, 1.7 mm and 3.5 mm, respectively, at each stage. Collapse occurred in the bolt anchorage zone of the roof at a pressure larger than 0.9 MPa, with the roof displacement reaching the maximum range of the sensor (15 mm) in an instant.

As shown in Fig. 16b, the sensors D3 and D4 were adopted to measure the displacement of the roof and the coal wall of Roadway ②, respectively. When the loading pressure reached 0.5 MPa, the surrounding rocks exhibited similar deformation characteristics with those of Roadway ①, except for a degree of variation that was more dramatic. During this process, the amount of roof subsidence was larger than the amount of coal wall deformation, which stood at 5.0 mm and 3.2 mm, respectively. At a loading pressure of 0.6 MPa, 0.7 MPa and 0.8 MPa, respectively, the roadway deformation presented an evolution characteristic of progressive increase, with the roof subsidence at each stage reaching 5.8 mm, 7.6 mm and 9.6 mm, respectively. When the loading pressure was at 0.9 MPa, the shallow rock mass of the roadway instantly collapsed, due to which the roof subsidence promptly reached the maximum range of the sensor. The coal wall convergence also reached 15 mm given a loading pressure of 1.0 MPa, implying that the deformation of the roof was larger than that of the coal wall under the short bolt support.

According to Fig. 16c, the sensors D5 and D6 were used to measure the displacement of the roof and the wall of Roadway ③, respectively. The roof subsidence was very close to the coal wall convergence at a loading pressure of 0.5 MPa, which were measured as 2.9 mm and 2.4 mm, respectively, after stabilization. When the loading pressure stood at 0.6 MPa and 0.7 MPa respectively, comparatively small deformation occurred in surrounding rocks, and the deformation of the roof and the coal wall only reached 4.0 mm and 3.4 mm, respectively. Given a loading pressure of 0.8 MPa, 0.9 MPa and 1.0 MPa respectively, comparatively active deformation took place in surrounding rocks, with the amount (in roof subsidence, for instance) reaching 5.5 mm, 7.9 mm and 10.4 mm, respectively. When the load reached 1.1 MPa, the coal walls were crushed and collapsed, due to which the coal wall convergence reached the maximum range of the sensor in an instant. Moreover, the overall roof sank as a result. This indicated that the deformation of the roof was significantly smaller than that of the coal wall under the high-strength long bolt support.

### Evolution of roadway surface displacement field

**Figure 17:**
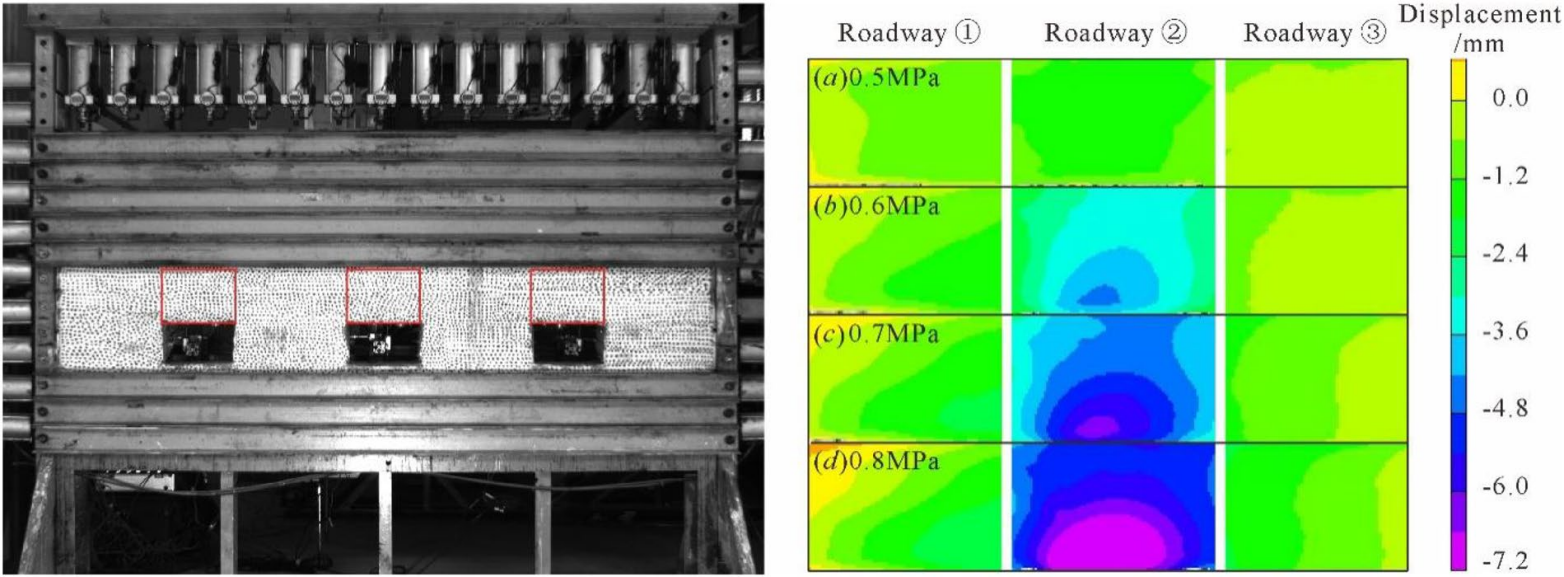
Evolution characteristics of displacement field of surrounding rock of each roadway under progressive loading.

Figure 17 presents the displacement field evolution diagram of the surrounding rocks of the roofs of the roadways under the mode of progressive loading. The digital speckle correlation measurement system was adopted to collect grayscale photos, which are shown as the images on the left in Fig. 17. The cloud chart characteristics of the roadway displacement fields were acquired after the input of the grayscale images into the software system for calculation, which are shown as the images on the right in Fig. 17.

**Figure 18:**
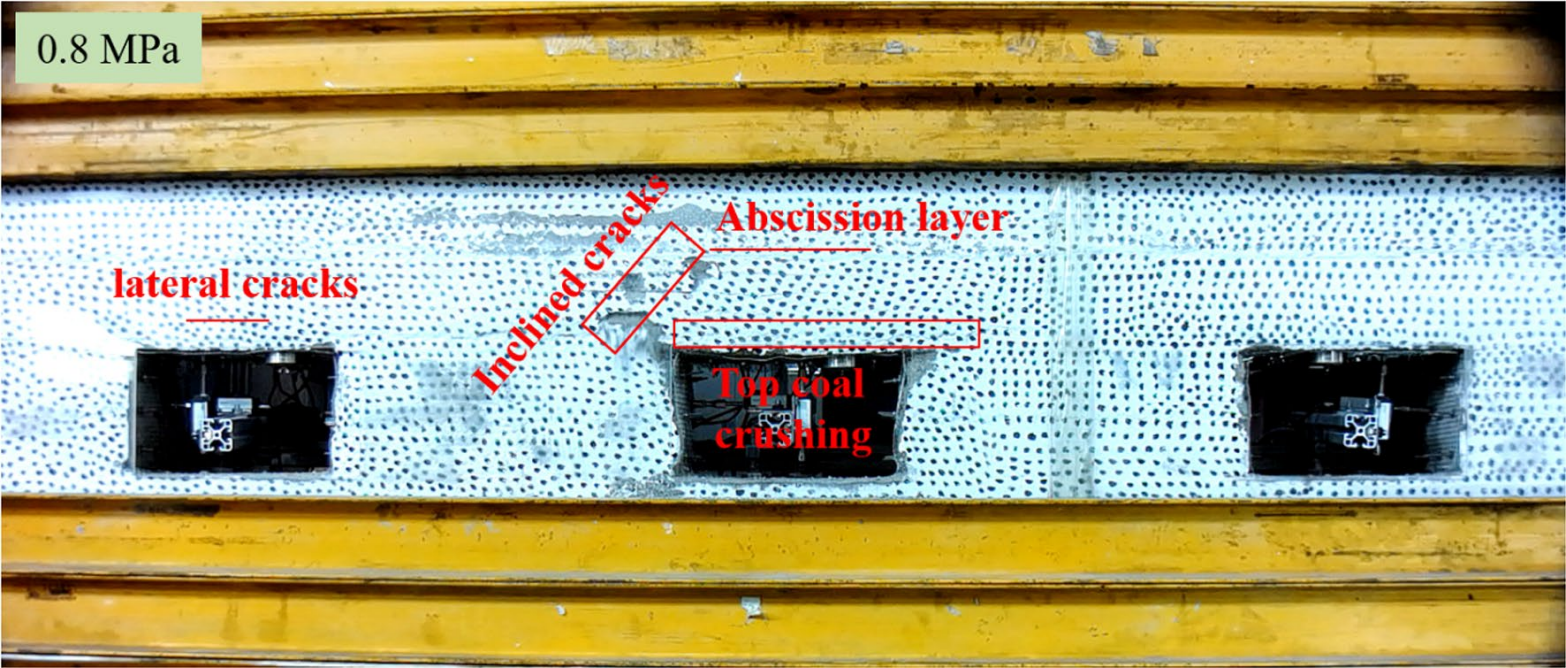
Failure characteristics of surrounding rock of each roadway at a loading pressure of 0.8 MPa.

According to Fig. 17a, the surrounding rock displacement fields of the roofs of all roadways remained relatively uniform when the loading pressure reached 0.5 MPa, without no concentration of displacement fields. Figure 17b reveals that the concentration of displacement fields occurred in the top coal area of Roadway ② and propagated into deeper strata when the loading pressure was at 0.6 MPa. On the contrary, however, no obvious changes were spotted in Roadway ① and Roadway ③ in this regard. As shown in Fig. 17c, the displacement field of the roof surrounding rock in Roadway ② continued to increase and propagated into deeper strata when the loading pressure reached 0.7 MPa. As shown in Fig. 17d, the displacement of the roof surrounding rock in Roadway ② distributed widely when the loading pressure was at 0.8 MPa, with the displacement of shallow surrounding rocks amounting to 7.2 mm. Nevertheless, no apparent concentration of displacement fields was observed in Roadway ① or Roadway ③. Further analysis of the roadway failure characteristics in Fig. 18 revealed extremely dramatic development of the cracks in surrounding rocks of Roadway ②, which not only generated inclined cracks and a lateral abscission layer but also caused the inclined cracks at the roadway humeral angle to intersect with and run through the abscission layer; the abscission layer was approximately located at the tail area of the bolt anchorage zone, falling into the tensile stress area between the bolts and the surrounding rocks and categorized as a weak surface. Therefore, it was easy for cracks to appear in this area under this load. At this moment, lateral cracks occurred between the top coal and the immediate roof of Roadway ①, while Roadway ③ only experienced minor deformation with the absence of apparent crack propagation.

## Discussions

### Difference analysis of progressive failures of surrounding rocks of different roadways

**Figure 19:**
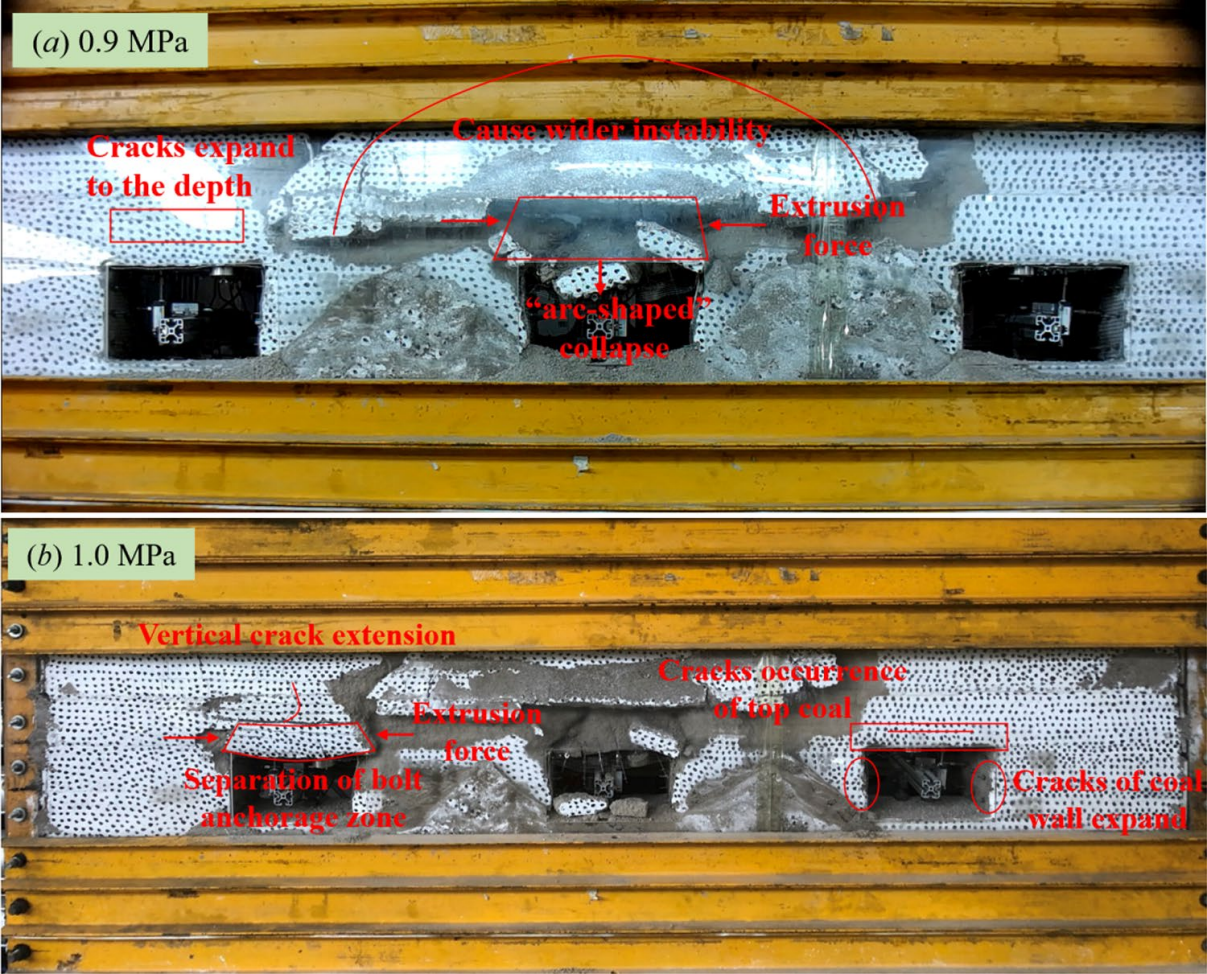
Failure characteristics of surrounding rocks of each roadway at a loading pressure of 0.9 MPa and 1.0 MPa.

Figure 19 presents the deformation failure diagrams of each roadway at a load of 0.9 MPa and 1.0 MPa, respectively. The failure mechanisms of the roofs anchored by different support systems were analyzed based on comparisons between failure characteristics of each roadway. As shown in Fig. 19a, overall collapse took place in Roadway ② when the load reached 0.9 MPa, subsequently resulting in instability of rock masses in a wider range. At this moment, though the cracks in shallow strata of Roadway ① had already propagated into deeper strata, the cracks were only confined to the bolt anchorage zone. On the contrary, however, Roadway ③ had witnessed no apparent deformation yet. The bolt anchorage zone of Roadway ② fell off further at a load of 1.0 MPa. In Roadway ①, the bolt anchorage zone was separated from the rock stratum of the immediate roof to cause complete failure of the bolt support. During this process, the cable only functioned to hang up the bolt anchorage zone. In the meanwhile, the vertical cracks in the immediate roof also propagated into deeper strata, so there was the risk of systematic collapse. At this moment, Roadway ③ also witnessed obvious deformation that mainly concentrated in the following two locations: firstly, the top coal and the connecting area between the top coal and the coal wall, where a lot of cracks appeared; and secondly, in the coal wall, where a great number of penetrating cracks occurred.

**Figure 20:**
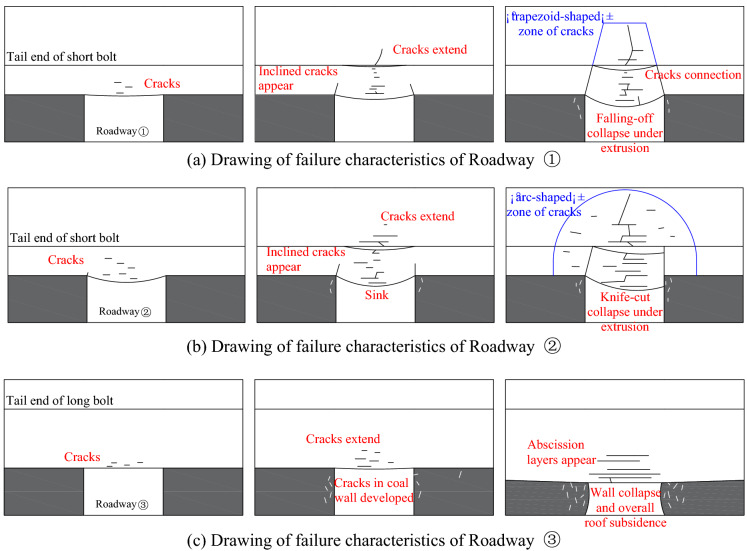
Drawings of failure characteristics of each roadway.

Figure 20a presents the drawing of the progressive failure of Roadway ①, which mainly consisted of the following processes: the cracks in the top coal started to emerge before the appearance of inclined cracks at the humeral angle in the connecting area between the top coal and the coal wall. As the lateral and longitudinal cracks in the anchorage zone ran through the strata, the overall bolt anchorage zone sank as a result and eventually fell off and collapsed under the action of the horizontal pressure force. This kind of failure is categorized as the “falling-off collapse under extrusion.” At this moment, the cracks had already propagated into the rock strata outside the bolt anchorage zone to form a “trapezoid-shaped” zone of cracks.

Figure 20b presents the drawing of the progressive failure of Roadway ②, which mainly covered the following processes: Cracks developed firstly in the top coal. Then, the cracks in the top coal propagated beyond the boundary to the entire bolt anchorage zone. In the meanwhile, the cracks at both the two humeral angles of the roadway started to develop upward, with inclined cracks formed on the left while vertical cracks on the right. Over collapse occurred in the bolt anchorage zone under the action of the horizontal force. This kind of failure is categorized as the “knife-cut collapse under extrusion.” At this moment, the cracks had already propagated beyond the bolt anchorage zone to form a wide “arc-shaped” zone of cracks.

Figure 20c presents the drawing of the progressive failure of Roadway ③, which mainly involved the following processes: The cracks on the top coal propagated into the immediate roof under high pressure. In the meanwhile, the cracks in the coal wall developed dramatically to trigger the collapse of the roadway and the overall subsidence of the roof. This is a failure typical of “wall collapse and overall roof subsidence.” During this process, the roadway still had safety spaces to some degree except for the appearance of multiple abscission layers in rock strata of the roof.

To sum up, the bearing mechanisms of the roof surrounding rocks of the three roadways differed from each other significantly. For Roadway ②, instead of playing a role in mobilizing the deep surrounding rocks to bear part of the load, the short bolts only functioned to construct a thin-layer anchoring structure in the roof, which was prone to collapse under the action of the load. For Roadway ①, the anchoring layer constructed by short bolts were reinforced by long cables to enhance the rigidity and strength of the bolt anchoring layer, thus preventing it from deformation. Under the action of high load, however, it would be easy for the cracks in the top coal to propagate and thus damage the integrity of the anchoring structure, which would cause a falling-off failure and reduce the bearing capacity of the cable. For Roadway ③, the flexible long bolts with high pre-stressing force contributed to the construction of a thick-layer anchoring structure in the roof, which could fully mobilize the deep surrounding rocks to bear part of the load. Minor displacement in deep strata was utilized to restrict major displacement in shallow strata, thus achieving the linkage of the displacement between deep and shallow strata. During this process, the thick-layer anchoring structure was typical of higher stiffness and strength and less prone to bending and deformation. With this structure, it would be hard for the cracks to propagate and run through the deep surrounding rocks.

### Exploration of impact resistance of thick-layer anchoring system in the roof of the roadway

**Figure 21:**
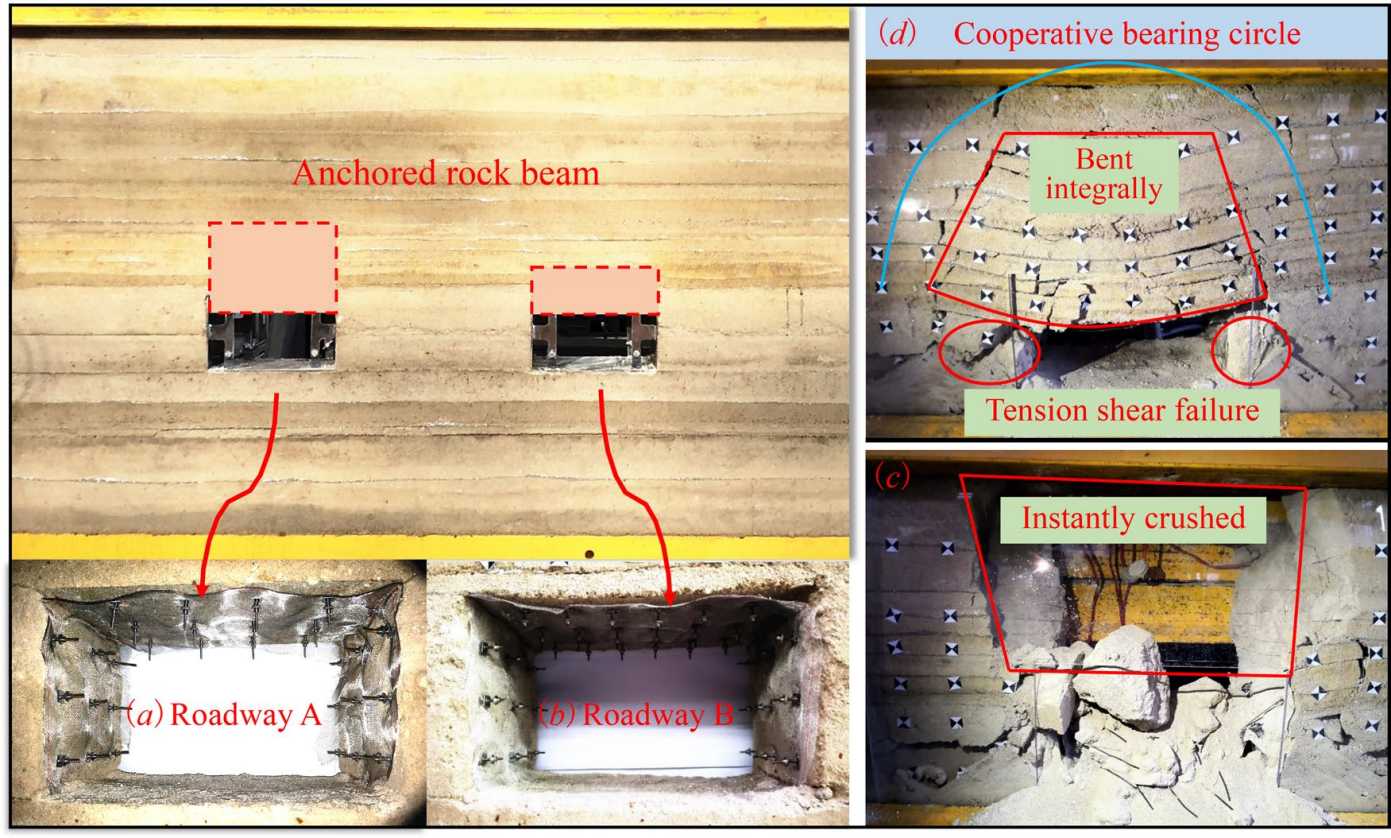
Roadway failure characteristics based on similarity model and impact dynamic load.

With Haulage Roadway 21,205 of Hulusu Coal Mine as the experimental background, a similar experimental model was prepared in line with the proportioning parameters in Table 1 to observe the maintenance and control effects of the roadways with anchoring layers of different thicknesses under high pressure. As shown in Fig. 21, two roadways were designed in the model to represent the thin-layer anchoring structure (Roadway A) under short bolt support (thickness of the anchoring layer: 100 mm) and the thick-layer anchoring structure (Roadway B) under long bolt support (thickness of the anchoring layer: 200 mm). Identical supports were adopted for the walls of the two roadways, both of which were designed as shown in Fig. 10.

After the supports for the roadways in the model were installed, the model was subjected to an instantaneous loading of 1.0 MPa to simulate the instantaneous high dynamic pressure under impact. The experimental results revealed significant differences in failure characteristics between the two roadways. According to Fig. 21c, the thin-layer anchored rock beam supported by short bolts in the roof of Roadway B collapsed in an instant under the action of the instantaneous high load, displaying the failure of “knife-cut” collapse in general. In the meanwhile, collapse outside the anchorage zone was also triggered. As shown in Fig. 21d, though the coal walls of Roadway A were instantaneously crushed under the action of high load, the thick-layer anchored rock beam in the roof only experienced “fan-shaped” subsidence as a whole. During this process, wide distribution of multiple horizontal and vertical cracks occurred between rock strata of the roof. At this moment, the failure of the roof was mainly attributed to the lack of bearing force from the coal wall.

Based on further analysis, the thickness of the anchoring layer played a key role in determining the bearing structure of the roof, thus further affecting the forms of the failures of the roadway. The thick-layer anchoring structure in the roof, supported by high-strength long bolts, could mobilize the rock masses in a wider range to bear the load together. In this manner, the rock masses functioned jointly to resist high load. Despite of the occurrence of wall collapse, the surrounding rocks of the roof could still bear the load effectively to guarantee necessary safety spaces for the roadway. Since the bearing capacity of the thin-layer anchoring structure supported by short bolts was weak, it could only mobilize the rock masses in a smaller range to bear the load. Consequently, it would instantaneously collapse under impact load at higher odds. This indicated that when a thick-layer anchoring structure has been established in the roof, it is able to effectively mobilize the rock masses in a wide range to form a huge bearing circle. In this way, the anchoring layer will be substantially improved in rigidity to perform better in impact resistance.

### Discussions on cases of cross-boundary support for deep coal roadways with composite roof

#### Comparisons between new and original support schemes

Haulage Roadway 21,205 of Hulusu Coal Mine was selected as the experimental site, which is a roadway with a rectangular section and is 5400 × 3200 mm in terms of specific size.

**Figure 22:**
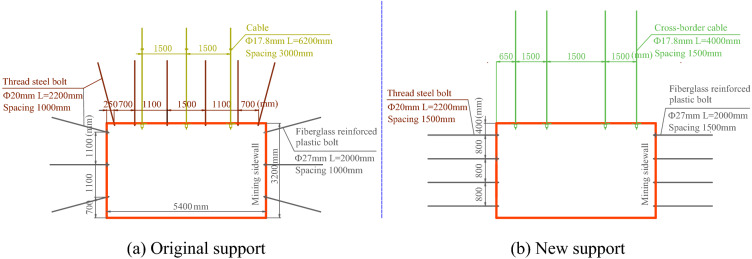
Comparison of support parameters between original and new schemes.

The original support scheme was a combination of bolt and cable support, with the specific parameters presented in Fig. 22a. Six thread steel bolts (specification: Ф20 × 2200 mm) were installed in each row in the roof, for which an arched steel supporting tray (150 × 150 × 10 mm) was equipped at a row spacing of 1000 mm. Three cables were installed (specification: Ф17.8 × 6200 mm) in each row, for which an arched steel supporting tray (300 × 300 × 12 mm) was arranged at a row spacing of 1500 × 3000 mm. On top of that, the installation of a steel bar mesh (specification: Ф6.5 × 3400 × 1100 mm) was also required. The pre-tightening force of the bolt and the cable was measured as 30 kN and 80 kN, respectively. Thread steel bolts (specification: Ф20 × 2200 mm) were adopted for the coal pillar, for which a steel supporting tray (150 × 150 × 10 mm) and a rhombic metal mesh (2800 × 1200 mm) were equipped. Fiberglass reinforced plastic bolts (Ф27 × 2000 mm) were adopted for the stoping wall, for which a fiberglass reinforced plastic supporting tray and a steel plastic mesh (2900 × 1200 mm) were equipped.

The new support scheme was a design of cross-boundary single support by cables, with the specific parameters presented in Fig. 22b. Cross-border cables (specification: Ф21.8 × 4000 mm) were adopted for the roof, with each row consisting of 4 such cables vertically at unequal spacing and a row spacing of 1500 mm. An arched steel supporting tray (300 × 300 × 16 mm) was equipped for each cable. A Ф6-mm steel bar mesh (5400 × 1600 mm) was also arranged to protect the surrounding rock surface of the roadway. The pretension force of the cable was measured as no less than 160 kN. Dextral high-strength thread steel bolts (specification: Ф20 × 2200 mm) and the alike were adopted for the coal pillar and the coal wall, for which a rhombic mesh (3300 × 1800 mm) was equipped to protect the surrounding rock surface of the roadway. Fiberglass reinforced plastic bolts (Ф27 × 2000 mm) were used for the stoping wall, for which a steel plastic mesh (3300 × 1800 mm) was installed for protection of the surrounding rock surface of the roadway. Four bolts were installed in each row at a row spacing of 800 × 1500 mm. The pre-tightening force of the two types of bolts was measured as no less than 40 kN.

#### Comparison of control effects on roadways between new and original schemes

**Figure 23:**
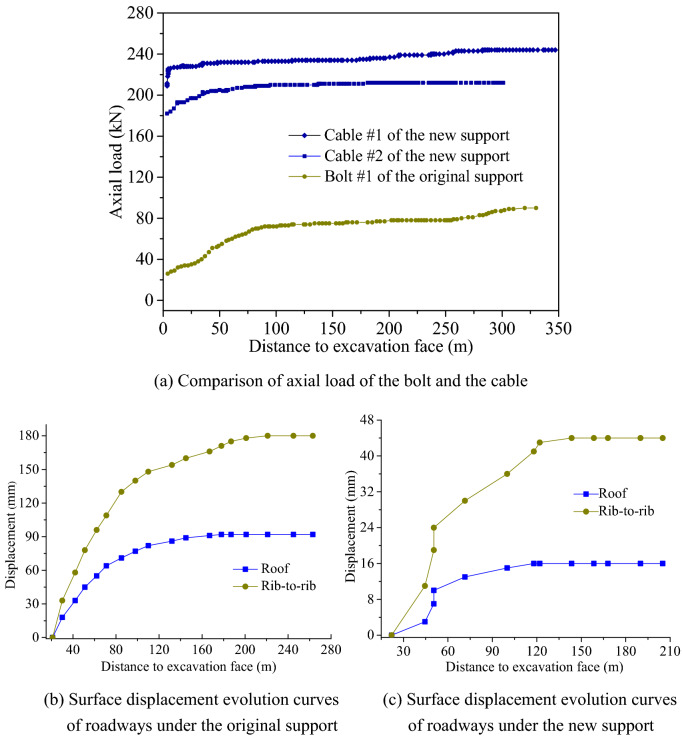
Monitoring data under the original support and the new support.

The axial load of the bolt and the cable and the surface deformation of the roadway anchored in line the original and new support schemes were monitored, respectively, using a dynamometer, with the monitoring results listed in the figure below. As shown in Fig. 23a, the initial anchor-hold of the bolt was only 26 kN under the original support, which was relatively small for immediate bearing of the load to constrain the comparatively large initial deformation of the roadway. In the meanwhile, the bolt load only rose slowly as the deformation of the roof aggravated. The load only increased to 36 kN when the excavation face was dug at a depth of 30 m. Abscission layers had already occurred in the top coal as the roof further sank, thus leading to substantial rise of the bolt load. When the excavation face was dug at a depth of 100 m, the bolt load had already reached 72 kN with an increase by 177%. Due to the small initial anchor-hold of the bolt, the bolt was able to work with a strong bearing capacity only when comparatively large deformation took place in the roof of the roadway. But the bolt anchorage zone was only 2.1 m thick at this moment. Therefore, it would be easy for the cracks to propagate beyond the bolt anchorage zone when the deformation of brittle rock masses continued, causing the risk of poor safety as a result. The initial anchor-hold of both cables was greater 180 kN under the new support. Therefore, the cables could function to restrict the initial deformation of the roofs as soon as slight deformation emerged. In general, the cable load rose sharply and then slowly and finally became stable. This tendency was more apparent when the cable load was further from the working face. Roof deformation was active within a range of 20 m from the working face. Instantaneous collapse occurred in the roof due to the excavation and unloading of the roadway, which explained the sudden rise of the cable load. At a distance 20 m away from the working face, the incremental load of the two cables accounted for 54.3% and 50.0% of the total incremental amount, respectively. The final load of the two cables stood at 244 kN and 212 kN, respectively. Since both of them were smaller than 45% of the peak load, it meant the stable working performance of the cables.

The roof subsidence and the convergence of both walls of the roadway under the original and new support schemes were monitored based on the cross-section method, with the monitoring results presented in Fig. 23b,c. The total influence scope resulting from excavation was about 200 m under the original support scheme. The final deformation of the roof and both walls was measured as 92 mm and 180 mm, respectively, with the scope subjected to severe influences being approximately 80 m. At this moment, the roof subsidence and the convergence of both walls accounted for 74% and 68% of the total deformation, respectively. It could be seen that the roadway experienced comparatively large surface deformation, and the roof sank to varying degrees. The top coal particularly witnessed severe deformation typical of broken expand. Moreover, a large area of fractured fissures occurred in the coal walls to result in the enlargement of roadway span and further aggravate the subsidence of the roof. The stress scope under the influence of excavation after adjustment was about 120 m under the new support scheme. In times of stable load, the roof subsidence and the convergence of both walls were measured as 15 mm and 44 mm, respectively. The first 50 m was the section where the excavation stress was adjusted dramatically. At this moment, the roof subsidence and the convergence of both walls stood at 11 mm and 25 mm and accounted for 73.3% and 56.8% of the total deformation, respectively. The roadway under the new support scheme generally witnessed not too much surface deformation. This was because the thick-layer bearing circle of the roof substantially inhibited the roadway from deformation, which not only adapted to energy absorption and alleviated the pressure from the roof on the coal wall but also controlled the problem of large-scale fragmentation of the coal wall^23,24^.

## Conclusion

In this paper, a large-scale similarity model was laid according to similar conditions of the coal-rock roadway with composite roof of Haulage Roadway 21,205 of Hulusu Coal Mine, based on which various monitoring systems were adopted to monitor the surrounding rock stress, surface deformation, bolt stress, surrounding rock deformation field, and other data of the roadway under three different support systems in a real time manner. In combination with the experimental results and the engineering application outcomes in the field, the conclusions were drawn as follows:

1. The roadway surrounding rocks under different support systems vary significantly in load bearing capacity. In terms of deformation resistance capacity, Roadway ③ > Roadway ① > Roadway ②, i.e., the single support of high-strength long bolts outperforms the support combining short bolts and long cables, both of which outperform the single support of short bolts.
2. Significant differences exist in the forms of the failures of coal-rock roadways with composite roof under different support systems. The thin-layer anchorage body, formed through the short bolt support, is prone to gradual fragmentation under the double action of the vertical and horizontal stress, which is categorized as the “falling-off failure under extrusion”. The roadway under the support combining bolts and cables witnesses failures firstly in the top coal and subsequently the propagation of the cracks at the humeral angle of the top coal beyond the coal-rock boundary into deeper surrounding rocks, followed by the occurrence of overall collapse under the load in the end. This kind of damage is categorized as the “knife-cut failure under extrusion”. The thick-layer anchoring structure, constructed through the high-strength long bolt support, can fully mobilize the deep surrounding rocks to bear the load together, so the roof is less prone to failures. The coal wall, however, is the first place to have failures, which will subsequently trigger the overall subsidence of the roof. This kind of damage is categorized as the “failure with wall collapse and roof subsidence”.
3. The initial anchor-hold and the length of bolts and cables bear direct correlations to the bearing capacity of surrounding rocks. Given a comparatively large initial ancho-hold, the bolts and the cables function timely to constrain the continuous deformation of the top coal. Coupled with the lock-in effect of long bolts on composite coal-rock strata, a thick-layer bearing structure can be formed in the roof. As verified in the intense dynamic load experiment, the high pretension thick-layer (HPTL) anchoring technology is able to resist the influences of strong impact. On the contrary, since the short bolt support and the bolt-cable combined support fail to meet the technical requirements of high pretension and thick-layer anchorage, neither of them can achieve the stability of coal-rock composite roofs in the long run.
4. Based on the feedbacks of industrial experimental outcomes, the initial anchor-hold of the bolts in the original support scheme was smaller than 30 kN. Given a distance of 30 m away from the excavation face, the increase of the bolt load only accounted for 18.5% of the total amount of increase, denoting that the bolts fail to control the deformation of the roadway in the long run. In terms of the scheme of HPTL technology, however, the initial anchor-hold of the cables was all greater than 180 kN. As a result, the roadway witnessed active deformation within 20 m from the excavation face. During this period, the increase of the cable load accounted for more than 50% of the total amount of increase, revealing that the cables are able to timely constrain the micro deformation resulting from the disturbance of excavation stress and can, therefore, maintain the stability of the coal-rock roadway with composite roof in the long run.

## Data Availability

All data generated or analysed during this study are included in this published article.
